# Integrated Molecular Profiling of Colorectal Cancer by Tumor Location: Evidence from a Real-World Cohort with Primary and Metastatic Samples

**DOI:** 10.3390/cancers18040666

**Published:** 2026-02-18

**Authors:** Andreea-Raluca Cozac-Szoke, Ovidiu Simion Cotoi, Ute Mauer, Konrad Steinestel, Annette Arndt

**Affiliations:** 1 Pathophysiology Department, George Emil Palade University of Medicine, Pharmacy, Science, and Technology of Targu Mures, 540142 Targu Mures, Romania; ovidiu.cotoi@umfst.ro; 2 Institute of Pathology and Molecular Pathology, Bundeswehrkrankenhaus Ulm, 89081 Ulm, Germany; utemauer@bundeswehr.org (U.M.); konradsteinestel@bundeswehr.org (K.S.); annette2arndt@bundeswehr.org (A.A.)

**Keywords:** colorectal cancer, tumor location, microsatellite instability, mismatch repair deficiency, tumor mutational burden, molecular profiling, HER2/neu, PD-L1, pan-TRK

## Abstract

Background/Objectives: Colorectal cancer (CRC) shows significant molecular diversity influenced by tumor location. Right- and left-sided CRCs differ in terms of microsatellite instability (MSI), mutational burden, and actionable biomarkers. This study aimed to characterize the clinicopathological and molecular features of CRC stratified upon tumor location. Methods: A consecutive series of CRC cases was retrospectively analyzed. Tissue samples were obtained from primary tumors (71%) or metastatic lesions (29%). All cases were evaluated by histopathology, immunohistochemistry (IHC), and targeted next-generation sequencing (NGS). Tumor location was assigned based on the primary tumor (43 right-sided and 35 left-sided cases). Results: Right-sided CRCs were more frequent in older patients and females and showed higher rates of deficient MMR (42% vs. 17%, *p* = 0.02), MSI-H (39% vs. 14%, *p* = 0.02), and high tumor mutational burden (TMB-high, ≥10 Mutations/Mb, 56% vs. 28%, *p* = 0.02). The most frequent pathogenic class 5 mutations were *TP53* (65%), *APC* (49%), and *KRAS* (44%). *APC* was the most frequently mutated gene in both pathogenic (class 5) and likely pathogenic (class 4) categories, with class 5 variants more common in left-sided tumors and class 4 variants predominating in right-sided tumors. *BRAF* mutations showed a statistically significant trend toward higher frequency in right-sided tumors (*p* = 0.05). HER2/neu overexpression (3+) was seen in 15% of patients, exclusively in MSS left-sided tumors. PD-L1 expression (CPS ≥ 1) was detected in 20% of patients, irrespective of location, and pan-TRK IHC was negative in all cases. The 29% of samples derived from metastatic lesions were predominantly MSS/pMMR (87%). Conclusions: Tumor location in CRC correlates with distinct molecular patterns. Right-sided tumors are associated with dMMR, MSI-H, and higher TMB, while left-sided CRCs display more ERBB2 alterations and class 5 *APC* mutations. The results highlight the importance of integrating tumor location into personalized molecular diagnostics and therapeutic planning for CRC patients.

## Simple Summary

Colorectal cancer is a biologically diverse disease, and growing evidence suggests that tumors arising on the right or left side of the colon are biologically and genetically distinct. These differences can influence both prognosis and treatment decisions. In this study, we examined tumor samples from 78 patients, including both primary tumors and metastatic lesions, to investigate the relationship between tumor location and key molecular features, such as microsatellite instability, mutation profiles, and expression of therapeutic targets. We found that right-sided tumors were more likely to have DNA repair deficiencies, a high number of specific mutations, and gene alterations that differ significantly from left-sided tumors. Understanding these patterns helps refine how we classify and treat colorectal cancer in clinical practice. Our findings highlight the need for location-informed molecular testing to support more personalized and effective cancer care.

## 1. Introduction

Colorectal cancer (CRC) represents a major public health burden in Europe, being the second leading cause of cancer-related death, with a persistently high mortality rate largely driven by late-stage diagnosis, molecular heterogeneity, limited access to precision therapies, and variable implementation of screening programs across countries [1,2].

CRC is now widely recognized as a biologically diverse disease, with one of the most clinically relevant distinctions being the anatomical location of the primary tumor [3]. Right-sided (proximal) and left-sided (distal) colorectal adenocarcinomas differ in their embryological origin, presentation, and molecular and immunological profiles [3,4].

Right-sided tumors are frequently associated with microsatellite instability (MSI-H) caused by deficient mismatch repair (dMMR), *BRAF* mutations, and increased PD-L1 expression, indicating a more immune-enriched microenvironment. In contrast, left-sided tumors exhibit chromosomal instability and common mutations in *TP53* and *APC*. They are more likely to be ERBB2-amplified or responsive to anti-EGFR therapies, particularly in cases with *RAS/BRAF* wild-type status. These location-specific differences have a significant impact on clinical practice, diagnosis, treatment selection, and patient stratification [3,4,5].

Advances in next-generation sequencing (NGS) and immunohistochemistry (IHC) have resulted in a more detailed understanding of CRC biology, allowing for comprehensive molecular profiling to find actionable mutations, gene fusions, and further biomarkers. This profiling is becoming increasingly crucial for guiding treatment, especially in advanced stages or metastatic disease, where targeted and immune-based therapies are considered [6]. In this context, the novelty of the present study lies in its real-world design, integrating molecular and immunohistochemical profiling of CRC across both primary tumors and metastatic lesions, thereby reflecting routine diagnostic practice and clinically relevant decision-making.

Several biomarkers are clinically relevant in the molecular stratification of CRC because of their prognostic and predictive significance. Among the most studied and actionable targets are microsatellite instability (MSI), MMR status, PD-L1 expression, ERBB2 amplification, and *NTRK* gene fusions. Each of these has a distinct role in guiding patient selection for immunotherapy or targeted therapy, and their distribution often varies based on the tumor’s anatomical location [7,8].

MSI-H and dMMR status, found in about 15% of CRCs, is more common in right-sided tumors, especially in early-stage disease. It is linked to a hypermutated phenotype, high tumor mutational burden (TMB), and a positive response to immune checkpoint inhibitors. Therefore, MSI and MMR testing are now standard for all newly diagnosed CRCs, serving both therapeutic and hereditary cancer screening roles [9,10].

PD-L1, while well-established in other malignancies, has a more limited role in CRC. Its expression is more common in MSI-H and dMMR tumors, often co-occurring with high immune cell infiltration. Although not used routinely as an isolated biomarker in CRC, PD-L1 IHC may complement MSI evaluation and is under investigation in ongoing clinical trials [11].

*ERBB2* amplification is found in 2–3% of CRCs, predominantly in left-sided, *RAS/BRAF* wild-type tumors. These tumors are typically resistant to anti-EGFR therapy but may respond to anti-HER2 targeted treatments, prompting testing in the metastatic setting. Routine assessment in early-stage disease is currently not recommended [12].

*NTRK* fusions, although rare (<1%), are highly actionable and most frequently occur in MSI-H/dMMR and *RAS/BRAF* wild-type tumors, particularly in the proximal colon. Detection via pan-TRK IHC, followed by molecular confirmation, can identify candidates for TRK inhibitor therapy, with remarkable clinical benefit in selected cases [12].

This study aimed to provide an integrated molecular and immunohistochemical characterization of colorectal adenocarcinomas, combining NGS, biomarker profiling (PD-L1, HER2/neu, pan-TRK), and MSI/MMR evaluation. Both primary tumors and metastatic lesions were included to comprehensively assess the distribution of genomic alterations and immune-related features according to tumor location (left vs. right colon), and to identify clinically relevant differences with implications for personalized treatment strategies.

## 2. Materials and Methods

### 2.1. Patient Selection and Study Design

A total of 78 patients with primary or metastatic colorectal adenocarcinoma who underwent biopsy or surgical resection and molecular testing between 2012 and 2025 at the Institute of Pathology, Bundeswehrkrankenhaus Ulm, were included in this study. Histopathological and molecular analyses were performed on either the primary tumor (55/78; 71%) or metastasis (23/78; 29%). Tumors were categorized according to the anatomical location of the primary tumor—right colon (55%) or left colon (45%)—regardless of whether the analyzed sample originated from the primary site or metastasis.

Clinical data were retrospectively collected from medical records and included patient age, sex, primary tumor localization, presence of metastases, and treatment history where available.

Histopathological reports were reviewed to document the pathological tumor–node–metastasis (pTNM) classification, tumor grade, and relevant histological features, including the predominant adenocarcinoma subtype and growth pattern. Tumor staging was assigned according to the 8th edition of the American Joint Committee on Cancer (AJCC) staging system [13]. Histological growth patterns in surgically resected specimens were classified following the 5th edition of the World Health Organization (WHO) Classification of Tumours [14].

Molecular analysis was performed in all cases using a next-generation sequencing (NGS) platform, which enabled comprehensive mutational profiling through the TruSight Oncology 500 (TSO 500) assay.

The study was conducted in accordance with the ethical standards outlined in the Declaration of Helsinki and was approved by the ethics committee of the University of Ulm (No. 162/13).

### 2.2. Immunohistochemistry

A representative formalin-fixed, paraffin-embedded (FFPE) tissue block containing a minimum of 20% viable tumor cells was selected for each case. Serial 4 μm sections were cut and mounted on charged slides for immunohistochemical (IHC) analysis. Tissue sections were deparaffinized in xylene and rehydrated through a graded ethanol series at room temperature. Primary antibody incubation was carried out for 30 min at room temperature, followed by washing and incubation with biotinylated secondary antibodies. Immunoreactivity was visualized using 3-amino-9-ethylcarbazole as the chromogen (Ventana OptiView DAB IHC Detection Kit, Ref: 760-700, Mannheim, Germany). IHC was performed using the Ventana BenchMark Ultra immunostainer (Roche, Mannheim, Germany), according to the manufacturer’s standardized protocols. The following prediluted monoclonal antibodies (Ventana, Roche) were employed: anti-HER2/neu (clone 4B5), PD-L1 (clone SP263), pan-TRK (clone EPR17341), anti-MLH1 (clone M1), anti-MSH2 (clone G219-1129), anti-MSH6 (clone SP93), and anti-PMS2 (clone A16-4). 

Loss of nuclear staining in tumor cells, with retained staining in adjacent non-neoplastic cells serving as internal controls, was interpreted as loss of expression (deficient MMR, dMMR). Preserved nuclear staining in tumor cells was considered proficient MMR (pMMR). Loss of MLH1 expression on immunohistochemistry was followed by confirmatory testing for *MLH1* promoter hypermethylation to differentiate between sporadic and germline dMMR.

HER-2/neu expression was evaluated according to the HERACLES diagnostic criteria, which consider both the intensity and distribution of membranous staining [15]. Cases scored as 2+ in ≥50% of tumor cells (equivocal) or 3+ in >10% and <50% of tumor cells underwent confirmatory *ERBB2* gene amplification testing by fluorescence in situ hybridization (FISH).

Pan-TRK immunoreactivity was defined as any staining above background intensity in ≥1% of tumor cells, regardless of the localization pattern (membranous, cytoplasmic, perinuclear, or nuclear).

The Combined Positivity Score (CPS) was calculated by dividing the number of PD-L1-positive cells (including tumor cells, lymphocytes, and macrophages) by the total number of viable tumor cells and then multiplying by 100. Non-neoplastic human tonsillar tissue served as a positive control. All IHC slides were independently reviewed by two pathologists.

### 2.3. Molecular Analysis

Genomic tumor DNA was extracted from microdissected FFPE tissue cuts using the Maxwell^®^ CSC DNA FFPE Kit on the Maxwell^®^ CSC Instrument (Promega, Madison, WI, USA). DNA concentration was measured using the Qubit 4 Fluorometer (Thermo Fisher Scientific, Waltham, MA, USA). DNA intended for NGS analysis was fragmented using the ME220 Focused ultrasonicator from Covaris (Brighton, UK).

Library preparation and enrichment were performed according to the manufacturer’s protocol for the Illumina TruSight Oncology 500 (TSO500) assay. Quality control of the library was done on the Agilent 5200 Fragment Analyzer (Agilent, Santa Clara, USA). Sequencing took place on the Illumina NextSeq 550Dx platform (Illumina, San Diego, USA). For data analysis, the onboard TruSight Oncology 500 Local App v2.0 was used. Variant classification and interpretation were done using the Molecular Health Guide database (Molecular Health GmbH, Heidelberg, Germany), as well as free versions of other databases (e.g., OncoKB™—MSK’s Precision Oncology Knowledge Base and JAX Clinical Knowledgebase) [16,17]. Variant classification into class 4 (likely pathogenic) and class 5 (pathogenic) was based on these molecular databases and established interpretation frameworks. This classification reflects the biological significance of the detected genomic alterations and does not automatically equate to clinically validated predictive or prognostic biomarker status.

For determination of the *MLH1*-promoter methylation status, the isolated DNA was bisulfite-converted using the EpiTect Bisulfite Kit (Qiagen) and analyzed by pyrosequencing using the PyroMark Q24 CpG *MLH1* Kit and the PyroMark Gold sequencing reagents on a PyroMark Q24 MDx instrument following the manufacturer’s protocols (Qiagen, Hilden, Germany). MSI status was assessed using a PCR-based assay evaluating microsatellite instability, in accordance with routine diagnostic protocols.

HER2/neu-fluorescence in situ hybridization (FISH) analysis was done using the ZytoLight^®^SPEC *ERBB2/CEN17* Dual Color probe mix on the ZytoBrite hybridizer ZytoVision (Bremerhaven, Germany). All steps were done exactly according to the manufacturer’s protocol. A case was scored positive when the *ERBB2*/*CEN17* ratio was ≥2.

### 2.4. Statistical Analysis

Categorical variables were summarized using absolute counts and percentages (n, %) and compared with Fisher’s exact test. Continuous variables are presented either as mean with standard deviation or median with interquartile range [IQR], based on their data distribution, and analyzed with Student’s *t*-test or the non-parametric Wilcoxon rank-sum test. All statistical tests were two-sided, with a *p*-value < 0.05 indicating significance. Analyses were conducted using GraphPad Prism (version 8, GraphPad Software, San Diego, CA, USA). Oncoplot and Heatmap were created in Microsoft Excel (Microsoft Office 365, version 2024).

## 3. Results

### 3.1. Clinicopathological and Population Characteristics

The average age of patients with right-sided CRC was 69 years, notably higher than that of patients with left-sided tumors (58 years; t = 2.99, *p* = 0.003). Interestingly, 17 patients (21%) were under 50 years old, qualifying as early-onset CRC. Right-sided CRC was significantly more common in female patients (61%), while left-sided tumors were mostly found in males (71%) (*p* = 0.006, OR = 3.82, 95% CI: 1.52–9.60).

Most samples came from primary tumors (*n* = 55, 71%) and were obtained through surgical resection (68%). A total of 29% of cases (*n* = 23) were biopsies from metastatic lesions. Among the 52 cases with complete TNM staging data, 12 patients (23%) presented with metastatic disease (M1), and 7 cases (58%) originated in the left colon. Of these, only 3 cases (25%) were classified as MSI-H, including two right-sided and one left-sided primary tumors.

The predominant histological subtype was adenocarcinoma not otherwise specified (NOS, 86%). Tumor grade and growth pattern were assessed in 52 cases, with G2 tumors (52%) and infiltrative patterns (52%) being the most frequent. Detailed case information is provided in Supplementary Table S1.

### 3.2. Driver Mutations and Mutation Frequencies

Among the 78 cases analyzed, the most frequently mutated genes (among class 5-pathogenic mutations) were *TP53* (65%), followed by *APC (49%)* and *KRAS* (*44%*). Other recurrent class 5 mutations included *BRAF* (22%), *PIK3CA* (17%), *BRCA2* (12%), *MLH1, PTEN*, *SMAD4* (6%), and *ATM* (5%). See Supplementary Figure S1 for an Oncoplot illustrating the distribution and frequency of common class 4 and class 5 mutations across the analyzed cohort.

Among class 4 (likely pathogenic) variants, *APC* was the most frequently mutated gene, identified in 40% of analyzed cases. Other recurrent class 4 mutations included *RNF43* (24%), *ARID1A* (17%), *FBXW7* (10%), and *SOX9* (10%). Additional alterations were observed in *B2M*, *SMAD4*, *BCORL1*, *CREBBP1*, and *ARID2*, each with frequencies ranging from 9% to 6% (Supplementary Figure S1).

When comparing mutation types by classification, missense mutations were significantly more common in class 5 than in class 4 variants (38% vs. 17.1%, *p* < 0.0001). Although splice-site mutations were relatively rare, they were more frequently classified as class 5 variants (6.6% vs. 1.4%, *p* = 0.001). Conversely, frameshift deletions and insertions were significantly more frequently classified as class 4 than class 5 mutations (41.2% vs. 17.6%, *p* < 0.0001 for deletions; 10.1% vs. 4.7%, *p* = 0.01 for insertions). Other mutation types (nonsense, in-frame deletions, etc.) showed no significant differences between the classes (Table 1 and Table 2).

**Figure 1:**
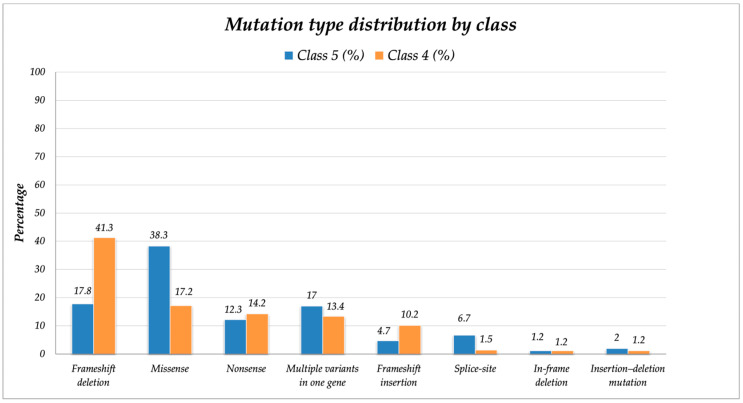
Distribution of mutation types according to variant pathogenicity class. The bar chart compares the relative frequency (%) of different mutation types between pathogenic variants (Class 5, orange) and likely pathogenic variants (Class 4, blue) identified in the CRC cohort. Missense and splice-site mutations were significantly more common in Class 5, whereas frameshift deletions and insertions predominated in Class 4. The other mutation types showed a more balanced distribution.

Multiple variants in the same gene occurred in 16.8% of class 5 and 13.3% of class 4 mutations (Figure 1).

This co-occurrence is also evident within the Oncoplot, where multiple mutations were present in individual cases (Supplementary Figure S1).

Comparative localization analysis between mutated genes revealed statistically significant differences. In the analysis of class 5 variants, *APC* emerged as the most frequently mutated gene in left-sided CRC (*p* = 0.04). In contrast, within class 4 variants, *APC* showed the highest mutation frequency in right-sided tumors (*p* = 0.005). On the other hand, *BRAF* mutations showed a statistically significant trend toward higher frequency in right-sided tumors (30% vs. 11; *p* = 0.057), while *RNF43* was significantly more frequent in left-sided tumors (*p* = 0.04) (Table 3). These associations are also visually supported by the heatmap in Supplementary Figure S2.

### 3.3. MSI and MMR Status

MMR protein expression was evaluated by IHC in all cases. Loss of expression indicating deficient MMR (dMMR) was identified in 24/78 cases (31%), most frequently involving concurrent loss of MLH1 and PMS2 (21/24; 88%). MSI testing confirmed MSI-H status in 22/78 cases (28%), and the two cases showing discordant results between MMR IHC and MSI testing were kept in the dataset without further reclassification. The remaining 56/78 (72%) cases were classified as MMR-proficient (pMMR) and microsatellite-stable (MSS). Of the 23 metastatic lesions in the cohort, only 3 (13%) were MSI-H, while the remaining 20 (87%) were MSS. *MLH1* promoter methylation was assessed in 18 of the 21 tumors with loss of MLH1/PMS2 expression, depending on tissue availability and routine testing indications. Among these, 78% (14/18) showed *MLH1* hypermethylation. Information is provided in Supplementary Table S1.

A significant association was observed between tumor location and MMR status. Among right-sided CRCs, 18/43 (42%) were dMMR, compared to 6/35 (17%) of left-sided tumors. This difference was statistically significant (*p* = 0.02; OR = 3.48, 95% CI: 1.20–10.12), indicating that right-sided tumors were nearly four times more likely to exhibit dMMR.

Among TMB-high tumors, 54% were MSI-H, while 46% were microsatellite-stable or MSI-low, indicating that high tumor mutational burden was not exclusively associated with MSI-H status.

Similarly, MSI-H status was more frequent in right-sided CRCs, occurring in 17/43 (39%) compared to 5/35 (14%) of left-sided tumors (*p* = 0.02; OR = 3.92, 95% CI: 1.27–12.11), as shown in Table 4.

### 3.4. HER2/neu Status

**Figure 2:**
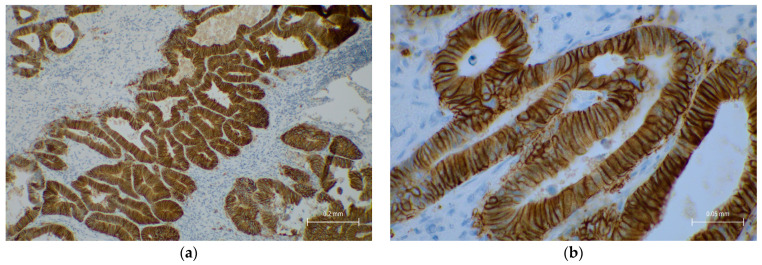
Representative HER2/neu IHC in a colorectal adenocarcinoma, scored as 3+ according to the HERACLES criteria. (**a**) Low-power field (original magnification ×100) showing strong, complete membranous staining in >50% of the tumor cells. (**b**) High-power view (original magnification ×400) confirming intense, circumferential membrane staining.

HER2/neu immunohistochemistry was performed in 71 cases. HER2/neu score 3+ was observed in 15%, score 2+ in 15%, score 1+ in 28%, and score 0 in 32% (Supplementary Table S1 and Figure 2).

A tendency toward a statistically significant association was identified between HER2/neu score 3 expression and tumor location (*p* = 0.07, OR = 0.32, 95% CI: 0.09–1.05), and all HER2/neu 3+ cases occurred in MSS tumors (Table 4).

**Figure 3:**
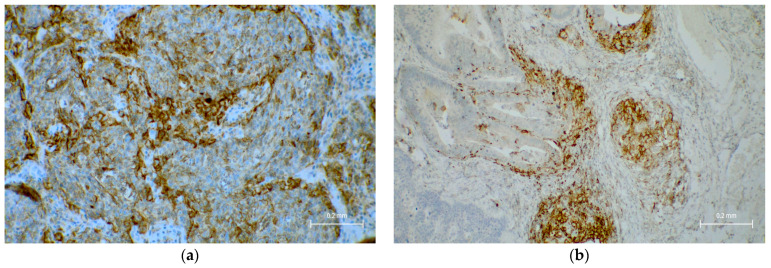
PD-L1 immunohistochemistry in a colorectal adenocarcinoma with CPS >1. (**a**) Membranous staining is observed in tumor cells, original magnification ×100, (**b**) along with scattered PD-L1-positive lymphocytes and macrophages within the tumor stroma, original magnification ×100.

PD-L1 expression (CPS ≥1) was identified in 20% of tumors (16/64). There was no significant association with tumor location (*p* = 0.78). Pan-TRK expression was negative in all cases (Table 4 and Figure 3).

TMB was distributed across five predefined categories: very low (7%), low–medium (19%), medium–high (28%), high (14%), and very high (32%). See Supplementary Figure S2 for a Heatmap summarizing molecular and clinicopathological characteristics stratified by tumor location. For analytical purposes, cases were grouped into a TMB-high group (high and very high, ≥10 mutations/Mb) and a TMB-low group (very low, low–medium, and medium–high, <10 mutations/Mb). Right-sided CRC showed a higher prevalence of TMB-high cases (56%, 24/43), compared to left-sided tumors (28%, 10/35), with a statistically significant association (*p* = 0.02, OR = 3.16, 95% CI: 1.22–8.15) (Table 4).

## 4. Discussion

This study aimed to characterize the clinicopathologic and molecular features of CRC stratified by anatomical location, incorporating IHC, MSI status, and targeted NGS data in a real-world hospital setting. Several of the observed patterns confirm well-established biological differences between right- and left-sided CRC cancer, including the enrichment of dMMR/MSI-H tumors and TMB in right-sided tumors, as well as the predominance of HER2 positivity in left-sided tumors.

In addition to these confirmatory findings, including both primary tumors and metastatic lesions provided a more accurate view of how CRC presents and develops in clinical practice. Since metastases can evolve and display changes in biomarker expression, their inclusion enhances the clinical relevance of our findings while also reflecting the biological complexity encountered in daily practice.

### 4.1. Clinicopathological and Demographic Characteristics

In this cohort, tumors were nearly evenly distributed between the right (55%) and left (45%) sides of the colon. Although population-based studies often report a predominance of left-sided CRCs, the balanced distribution observed here reflects real-world patterns seen in mixed clinical populations. Importantly, this near-equal distribution provides a methodological advantage, as it allows for a more robust comparison between the two tumor locations. Also, it emphasizes the increasing trend in the detection of proximal tumors, particularly in screening-naïve populations [18].

A statistically significant association was observed between tumor location and patient sex. Right-sided tumors were more frequent among females (61%), while left-sided tumors predominated among males (71%) (*p* = 0.006; OR = 3.82). Similar patterns have been described in other studies and may point to underlying biological influences, possibly hormonal or immune-mediated, in tumor development and localization [18].

Histologically, most tumors were conventional adenocarcinomas (NOS, 86%). Tumor grade was mainly G2 (52%), and the growth pattern was nearly evenly split between infiltrative and expansive types, consistent with other studies. However, none of these features showed a significant association with tumor location or sex in this dataset [17].

### 4.2. Mutation Landscape and Molecular Profiles

While class 4 and class 5 variants reflect biologically significant genomic alterations, only a subset currently qualify as validated predictive or prognostic biomarkers in colorectal cancer. The mutational landscape observed in this cohort reflects established features of CRC biology while also offering a few distinct patterns worth highlighting. Among class 5 variants, *TP53* mutations were the most frequent (65%), consistent with its central role in regulating cell cycle arrest and genomic stability. Mutations in *APC* (49%) and *KRAS* (44%) followed closely, with both genes being key drivers of CRC in the early stages through the MAPK and WNT signaling pathways [19,20].

*APC* also emerged as the most frequently mutated gene among class 4 variants (40%). Alongside *APC*, other recurrent class 4 mutations included *RNF43* (24%), *ARID1A* (17%), *FBXW7* (10%), and *SOX9* (10%). While formally categorized as likely pathogenic, these variants occur in genes with well-established driver roles in colorectal cancer, supporting their functional significance. The presence of *FBXW7* and *SOX9* alterations further suggests that defects in cell-cycle control and differentiation programs contribute to tumor progression in this cohort [20,21].

A few genes with lower mutation frequencies, such as *B2M, SMAD4, BCORL1, CREBBP1*, and *ARID2*, were also detected in 9% to 6% of cases. While individually less common, their presence underscores the molecular diversity that accumulates as tumors progress. Notably, the detection of multiple mutations within the same gene in a subset of cases might suggest intratumoral heterogeneity, a feature known to complicate therapeutic decisions and the interpretation of predictive biomarkers. Importantly, some of these less frequent genomic alterations correspond to targets currently explored in precision oncology, highlighting their potential clinical relevance in later lines of therapy. This aspect becomes especially relevant in the context of metastatic lesions, which made up over a quarter of our cohort. Since metastases may show distinct evolutionary profiles compared to primary tumors, capturing their molecular features offers a more complete view of disease biology and therapeutic vulnerabilities [22,23].

Notably, the type of mutations differed between classes. Class 5 variants were mostly missense mutations, often associated with gain-of-function effects in oncogenes and loss-of-function effects in tumor suppressor genes. Although less frequent overall, splice-site mutations were also more common in class 5 than in class 4, suggesting that a subset of pathogenic alterations may act through disrupted RNA processing rather than classical amino acid substitutions. In contrast, in our cohort, class 4 variants more frequently involved frameshift deletions and insertions, suggesting loss-of-function mechanisms more typical of tumor suppressors. These differences align with the distinct roles these classes of genes tend to play, with class 5 mutations driving oncogenic signaling and class 4 alterations contributing to loss of genomic control or immune modulation [24].

The observed distribution also varied by tumor location in our cohort. The most pronounced side-related differences were seen in Wnt pathway genes. Pathogenic *APC* mutations were more frequent in left-sided tumors, whereas likely pathogenic *APC* variants predominated in right-sided CRCs, reflecting different patterns of Wnt pathway disruption. One plausible explanation is that distal CRCs more often follow the “classical” chromosomal instability pathway, in which pathogenic *APC* mutations drive the adenoma–carcinoma sequence. By contrast, proximal tumors, which are more frequently hypermutated or dMMR, may accumulate additional, lower-confidence *APC* alterations on a background of greater genomic instability. *RNF43* class 4 mutations were also more frequent in left-sided tumors, suggesting that distal cancers can activate Wnt signaling not only through *APC* but also through the inactivation of negative regulators such as *RNF43*.

Conversely, *BRAF* class 5 mutations showed a tendency toward higher frequency in right-sided tumors, consistent with the well-established association between *BRAF*-mutated CRC and proximal location [25].

### 4.3. MMR, MSI Status, and TMB Relationship

Our findings regarding MMR and MSI status are in line with known molecular patterns in CRC, but they also offer some distinct insights, especially given the heterogeneity of our cohort, which included both primary tumors and metastatic lesions.

Mismatch repair deficiency (dMMR) was identified in 31% of cases, while MSI-H status was identified in 28% of cases; the two cases exhibiting discrepancies is consistent with the recognized analytical and biological limitations of these assays, including subclonal protein loss and low tumor cell content. Most dMMR tumors involved concurrent loss of MLH1 and PMS2, and most tested cases showed *MLH1* promoter hypermethylation, pointing toward a sporadic rather than hereditary origin [26,27]. Among the MSI-H/dMMR tumors, 4 cases lacked *MLH1* promoter hypermethylation. In these cases, a hereditary MMR defect (Lynch syndrome) cannot be excluded, and the patients were referred for genetic counselling and germline testing [26,27].

As expected, MSI-H and dMMR phenotypes were significantly more common in right-sided tumors. Patients with tumors originating in the right colon were nearly four times more likely to show dMMR (OR = 3.48, *p* = 0.02) and four times more likely to have MSI-H status (OR = 3.92, *p* = 0.02) than those with left-sided tumors. These differences reflect well-established biological contrasts between proximal and distal CRCs and may also explain the differential response to immunotherapy observed in clinical settings [22].

One of the more interesting aspects of our data concerns the relatively low frequency of MSI-H in metastatic lesions. Among the 23 samples taken from metastases, only 3 (13%) were MSI-H. Moreover, in the 52 patients for whom complete TNM staging was available, 12 were classified as M1, and just 3 of the primary tumors (25%) had MSI-H. The rest of the primary tumors with M1 were MSS. This pattern aligns with the idea that MSI-H tumors tend to have a lower propensity to metastasize or may progress more slowly due to increased immune recognition [28].

Moreover, the distribution of metastatic disease was balanced between right- and left-sided tumors (5 vs. 7 cases). This suggests that while MSI and MMR status differ clearly by tumor location, metastatic potential may be more evenly distributed, at least in our cohort [29].

Importantly, the inclusion of both primary and metastatic samples was intentional and aimed at reflecting real-world clinical practice. Tumor location was defined according to the anatomical site of the primary tumor, even when molecular profiling was performed on metastatic tissue, to ensure consistent biological classification. This approach mirrors routine oncology practice, where therapeutic strategies are generally guided by the primary tumor origin, even when treatment decisions rely on molecular testing performed on metastatic lesions.

At the same time, it is well recognized that molecular features such as MMR and MSI status may differ between primary tumors and their metastases, particularly after systemic therapy. This phenomenon, often described as clonal evolution or clonal switching, reflects dynamic tumor biology under selective pressure. Rather than representing a methodological inconsistency, the inclusion of metastatic samples therefore captures this biological complexity and provides a more realistic representation of clinical decision-making in advanced colorectal cancer [30,31].

Given the known relationship between dMMR and hypermutation, we also evaluated TMB in parallel with MSI and MMR status. TMB showed considerable variability across the cohort. When dichotomized, TMB-high status (≥10 mutations/Mb) was observed with a significant predominance in right-sided tumors (56% vs. 28%, *p* = 0.02, OR = 3.16). This enrichment of high TMB in right-sided CRC supports its association with hypermutated phenotypes, including dMMR/MSI-H, and may have therapeutic relevance in the setting of immunotherapy. Still, the overlap is not complete, and high TMB can occasionally occur in MSS tumors, underlining the need for integrated biomarker evaluation [32,33].

### 4.4. HER2/neu, PD-L1, and pan-TRK Expressions

HER2/neu expression, PD-L1 status, and pan-TRK results also provided additional insights into the molecular landscape of our cohort. HER2/neu overexpression (score 3+) was identified in only 15% of cases, with a trend toward statistical significance in left-sided tumors (*p* = 0.07). All HER2/neu 3+ tumors were MSS, which aligns with the literature suggesting that HER2-driven CRCs are typically MSS and follow the CIN pathway. This molecular profile is clinically important, as HER2-targeted therapies are being explored in patients with MSS, *RAS/BRAF* wild-type, left-sided tumors [7,34].

PD-L1 expression (CPS ≥1) was present in 20% of tumors. However, it did not show a statistically significant correlation with tumor sidedness or other clinicopathologic parameters. While PD-L1 alone is not yet a definitive biomarker in CRC, its co-expression with MSI-H or high TMB may support immune-based therapeutic decisions in select cases [35,36].

As for pan-TRK, IHC was negative in all tested tumors. While this result was expected, given the rarity of *NTRK* fusions in CRC populations, pan-TRK IHC remains a valuable screening tool due to its cost-effectiveness and broad accessibility. When positive, IHC can serve as a practical first-line method to flag potential fusion-positive tumors, which can then be confirmed by more specific techniques such as RNA-based NGS. In this way, pan-TRK IHC supports a rational and resource-conscious approach to identify candidates for TRK inhibitor therapies [12,37].

This study has several limitations that should be acknowledged. First, its single-center and retrospective design may limit the generalizability of the findings. Second, the cohort size and the absence of longitudinal follow-up data precluded survival analyses and assessment of the prognostic impact of the investigated biomarkers.

## 5. Conclusions

This study offers a real-world perspective on the molecular and immunohistochemical landscape of CRC, encompassing both primary and metastatic samples and capturing a broader spectrum of disease biology.

Our findings confirm known trends, such as the higher prevalence of MSI-H/dMMR in right-sided tumors and the dominance of *APC* and *BRAF* mutations in advanced stages. HER2/neu overexpression was observed only in MSS tumors and occurred more frequently in left-sided CRC, supporting its relevance as a therapeutic marker in this subgroup.

The inclusion of metastatic lesions allowed us to highlight the low MSI-H rate in metastases and the presence of potential intratumoral heterogeneity. PD-L1 expression (CPS ≥ 1) showed no clear association with tumor location, supporting its use as a complementary rather than independent biomarker. Finally, our results support pan-TRK IHC as a cost-effective tool for initial screening of *NTRK* fusions, when followed by confirmatory RNA-based testing.

Altogether, this integrated approach reflects the complexity of real-life diagnostics and may help refine therapeutic strategies in CRC.

## Figures and Tables

**Table 1:** Most frequently mutated genes and their associated mutation types in the colorectal cancer cohort.

	Pathogenic Mutations (Class 5)				Likely Pathogenic Mutations (Class 4)			
No.	Gene	Mutation Type	Absolute No.	Frequency (%)	Gene	Mutation Type	Absolute No.	Frequency (%)
1	*TP53*		51	65	*APC*		31	40
2	*APC*		38	49	*RNF43*		19	24
3	*KRAS*		34	44	*ARID1A*		13	17
4	*BRAF*		17	22	*FBXW7*		8	10
5	*PIK3CA*		13	17	*SOX9*		8	10
6	*BRCA2*		9	12	*B2M*		7	9
7	*MLH1*		5	6	*SMAD4*		7	9
8	*PTEN*		5	6	*BCORL1*		6	8
9	*SMAD4*		5	6	*CREBBP1*		6	8
10	*ATM*		4	5	*ARID2*		5	6
**LEGEND**								
	
	
	
	
	

Missense substitution (blue); Multiple variants in one gene (black); Nonsense substitution (brown); Frameshift insertion (light brown); Frameshift deletion (green).

**Table 2:** Mutation type frequencies comparison: class 4 vs. class 5.

Mutation Type	Pathogenic Mutations n = 255	Likely Pathogenic Mutations n = 344	Fisher’s *p*-Value	OR	95% CI
Missense	97 (38%)	59 (17.1%)	<0.0001	2.97	2.03 to 4.33
Nonsense	31 (12.1%)	49 (14.2%)	0.46	0.83	0.51 to 1.35
Splice-site	17 (6.6%)	5 (1.4%)	0.001	4.84	1.76 to 13.31
In-frame deletion	3 (1.1%)	4 (1.1%)	0.99	1.01	0.22 to 4.56
Frameshift deletion	45 (17.6%)	142 (41.2%)	<0.0001	0.30	0.21 to 0.45
Frameshift insertion	12 (4.7%)	35 (13.7%)	0.01	0.44	0.22 to 0.86
Multiple variants in one gene	43 (16.8%)	46 (13.3%)	0.24	1.31	0.84 to 2.06
Insertion–deletion mutation	5 (1.9%)	4 (1.1%)	0.50	1.70	0.45 to 6.40

OR = Odds Ratio; CI = Confidence Interval.

**Table 3:** Gene-level comparative analysis between left-sided and right-sided CRC (classes 5 and 4).

Gene	Right-Sided CRC (n = 43)	Left-Sided CRC (n = 35)	Fisher’s *p*-Value	OR	95% CI
Class 5					
*TP53*	28 (65%)	23 (66%)	0.99	1.03	0.40 to 2.62
*APC*	16 (37%)	22 (63%)	0.04	2.86	1.13 to 7.19
*KRAS*	18 (42%)	16 (46%)	0.82	1.17	0.48 to 2.88
*BRAF*	13 (30%)	4 (11%)	0.057	0.30	0.09 to 1.02
*PIK3CA*	8 (19%)	5 (14%)	0.763	0.73	0.22 to 2.47
Class 4					
*APC*	20 (48%)	11 (31%)	0.005	3.88	1.49 to 10.11
*RNF43*	3 (7%)	16 (46%)	0.003	0.16	0.04 to 0.60
*ARID1A*	4 (9%)	9 (26%)	0.3634	0.49	0.14 to 1.74

OR = Odds Ratio; CI = Confidence Interval.

**Table 4:** Tumor location-associated differences in MMR status, *HER2/neu*, and PD-L1 expression.

Parameter	**Right-Sided CRC (n = 43)**	**Left-Sided CRC (n = 35)**	**Fisher’s *p*-Value**	**OR**	**95% CI**
MSI-H vs.	17 (39%)	5 (14%)	0.02	3.92	1.27–12.11
MSS	26 (61%)	30 (86%)			
dMMR vs.	18 (42%)	6 (17%)	0.02	3.48	1.20–10.12
pMMR	25 (58%)	29 (83%)			
TMB			0.02	3.16	1.22–8.15
TMB-high group	24 (56%)	10 (28%)			
TMB-low group	19 (44%)	25 (72%)			
HER2/neu score (n = 71)					
Score 0	14 (35%)	11 (35%)	0.07	0.32	0.09–1.05
Score 1+	12 (30%)	10 (32%)			
Score 2+	10 (35%)	2 (6%)			
Score 3+	4 (10%)	8 (26%)			
PD-L1 (n = 64)			0.78	0.85	0.30–2.35
CPS ≥ 1	8 (23%)	8 (27%)			
CPS < 1	26 (77%)	22 (73%)			

OR = Odds Ratio; CI = Confidence Interval.

## Data Availability

The data presented in this study are available on reasonable request from the corresponding author. The data are not publicly available due to institutional privacy restrictions.

## Supplementary Materials

The following supporting information can be downloaded at: https://www.mdpi.com/article/10.3390/cancers18040666/s1, Table S1: Descriptive characteristics of the study population with CRC; Figure S1: Heatmap summarizing molecular and clinicopathological characteristics stratified by tumor location.; Figure S2: Oncoplot illustrating the distribution and frequency of common class 4 and class 5 mutations across the analyzed cohort.

## Abbreviations

The following abbreviations are used in this manuscript:

AJCC	American Joint Committee on Cancer
CIN	Chromosomal Instability
CPS	Combined Positivity Score
CRC	Colorectal Cancer
dMMR	Deficient Mismatch Repair
EGFR	Epidermal Growth Factor Receptor
FFPE	Formalin-Fixed Paraffin-Embedded
FISH	Fluorescence In Situ Hybridization
HER2	Human Epidermal Growth Factor Receptor 2
IHC	Immunohistochemistry
MMR	Mismatch Repair
MSI	Microsatellite Instability
MSI-H	MSI-High
MSI-L	MSI-Low
MSS	Microsatellite Stable
NGS	Next-Generation Sequencing
NOS	Not Otherwise Specified
NTRK	Neurotrophic Tyrosine Receptor Kinase
PCR	Polymerase Chain Reaction
PD-L1	Programmed Death Ligand 1
TMB	Tumor Mutational Burden
TSO 500	TruSight Oncology 500
WHO	World Health Organization
