# Supplementary material for: Integrated Molecular Profiling of Colorectal Cancer by Tumor Location: Evidence from a Real-World Cohort with Primary and Metastatic Samples
Source: Cancers (Basel). 2026 Feb 18;18(4):666. doi: 10.3390/cancers18040666 (PMC12939332; doi:10.3390/cancers18040666)
Supplement: Supplementary file 1 [file cancers-18-00666-s001.zip › Table S1.pdf]

Table 1. Descriptive characteristics of the study population with CRC

Parameter	*Total no. of cases n = 78*	
	n	%
**Gender**		
Males	42	54
Females	36	46
**Sample origin**		
Primary tumor	55	71
Metastasis	23	29
**Specimen type**		
Biopsy	25	32
Resection specimen	53	68
**Primary tumor localization**		
Right colon	43	55
Left colon	35	45
**KRAS status**		
KRAS mutation	34	44
KRAS wild-type	44	56
**NRAS status**		
NRAS mutation	1	1
NRAS wild-type	77	99
**BRAF status**		
V600E	17	22
BRAF wild-type	61	78
**MMR protein expression – IHC (n=78)**		
dMMR	24	30
Loss of MLH1+PMS2	21	27
Loss of MSH2+MSH6	1	1
Isolated loss of MSH6	1	1
Isolated loss of PMS2	1	1
MMR proficient	54	70
**MSI testing (n=78)**		
MSS	56	72
MSI-H	22	28
**MLH-1 methylation (n=18)**		
Non-methylated	4	22
Methylated	14	78

**Primary tumor histology**		
**Major histology subtype (n=78)**		
ACC NOS	67	86
Mixed ACC	5	6
Mucinous ACC	1	1
Medullary ACC	2	3
NEC	1	1
MiNEN	1	1
HG-IEN	1	1
**Tumor growth pattern (n=52)**		
Infiltrative	27	52
Expanding	25	48
**Histological grade (n=52)**		
G1 (well differentiated)	5	10
G2 (moderately differentiated)	27	52
G3 (poorly differentiated)	20	38
***HER2/neu* status**		
***HER2/neu* score (immunohistochemistry) (n=71)**		
0	25	35
1+	22	31
2+	12	17
3+	12	17
**FISH analysis of *HER2/neu (ERBB2)* gene (n=12)**		
Amplified	0	0
Non-amplified	12	100
**pan-TRK immunohistochemistry (n=64)**		
Positive	0	0
Negative	64	100
**PD-L1 immunohistochemistry (n=64)**		
CPS ≥ 1	16	20
CPS < 1	48	75
**Depth of invasion (pT stage) (n=52)**		
pT1	4	8
pT2	7	13
pT3	31	60
pT4	10	19
**Lymph node status (pN stage) (n=52)**		
pN0	26	50
pN1(a/b/c)	15	29
pN2(a/b)	11	21
**Distant metastasis (pM stage) (n=52)**		

pM0<br>pM1	40<br>12	77<br>23
**Lymphatic invasion (n=52)**<br>Present<br>Absent	 32<br>20	 62<br>38
**Venous invasion (n=52)**<br>Present<br>Absent	 7<br>45	 14<br>86
**Perineural invasion (n=52)**<br>Present<br>Absent	 9<br>43	 17<br>83
**Surgical margins (n=52)**<br>Positive<br>Negative	 8<br>44	 15<br>85

***** Descriptive clinicopathologic and molecular characteristics of the colorectal cancer study cohort. The table summarizes demographic, histopathologic, and molecular parameters for the entire cohort (n = 78), including both primary tumors and metastatic lesions**.** Variables include sample origin, localization, histological subtype, biomarker status (*KRAS, NRAS, BRAF,* MSI/MMR*, HER2/neu*, PD-L1, *pan-TRK*), and key pathological features such as invasion patterns, staging, and response to neoadjuvant therapy. Where applicable, subgroup sizes (n) are indicated.
