# Supplementary figures and images for: Integrated Molecular Profiling of Colorectal Cancer by Tumor Location: Evidence from a Real-World Cohort with Primary and Metastatic Samples
Source: Cancers (Basel). 2026 Feb 18;18(4):666. doi: 10.3390/cancers18040666 (PMC12939332; doi:10.3390/cancers18040666)

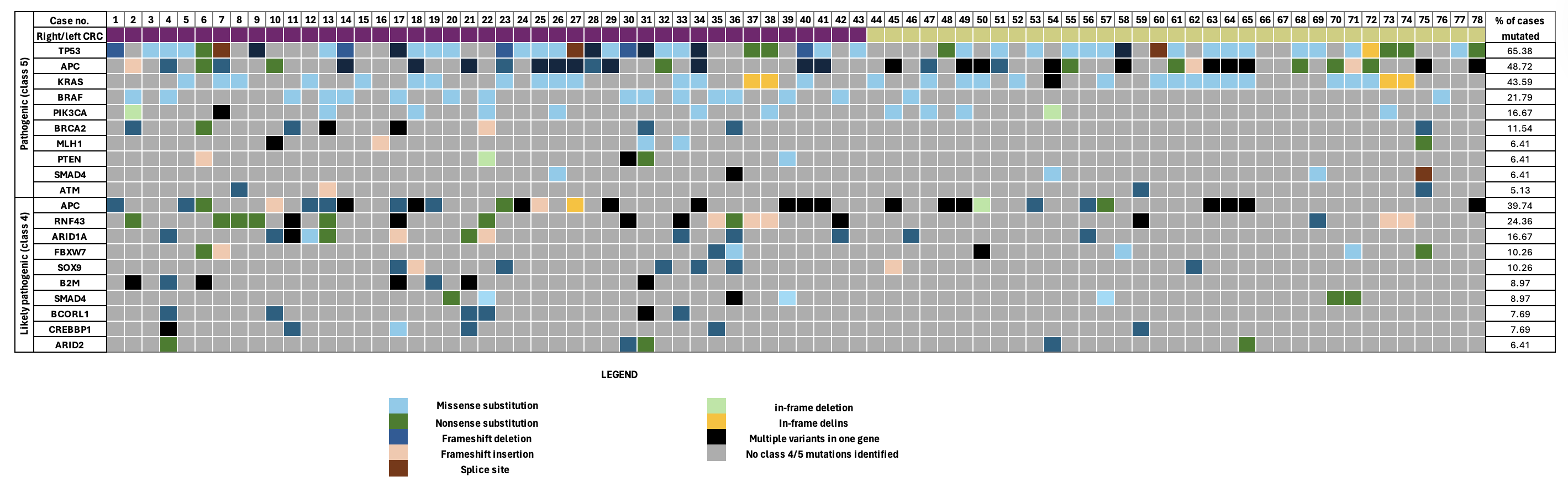

Supplement: Supplementary file 1 [file cancers-18-00666-s001.zip › Figure S1. Oncoplot.png]

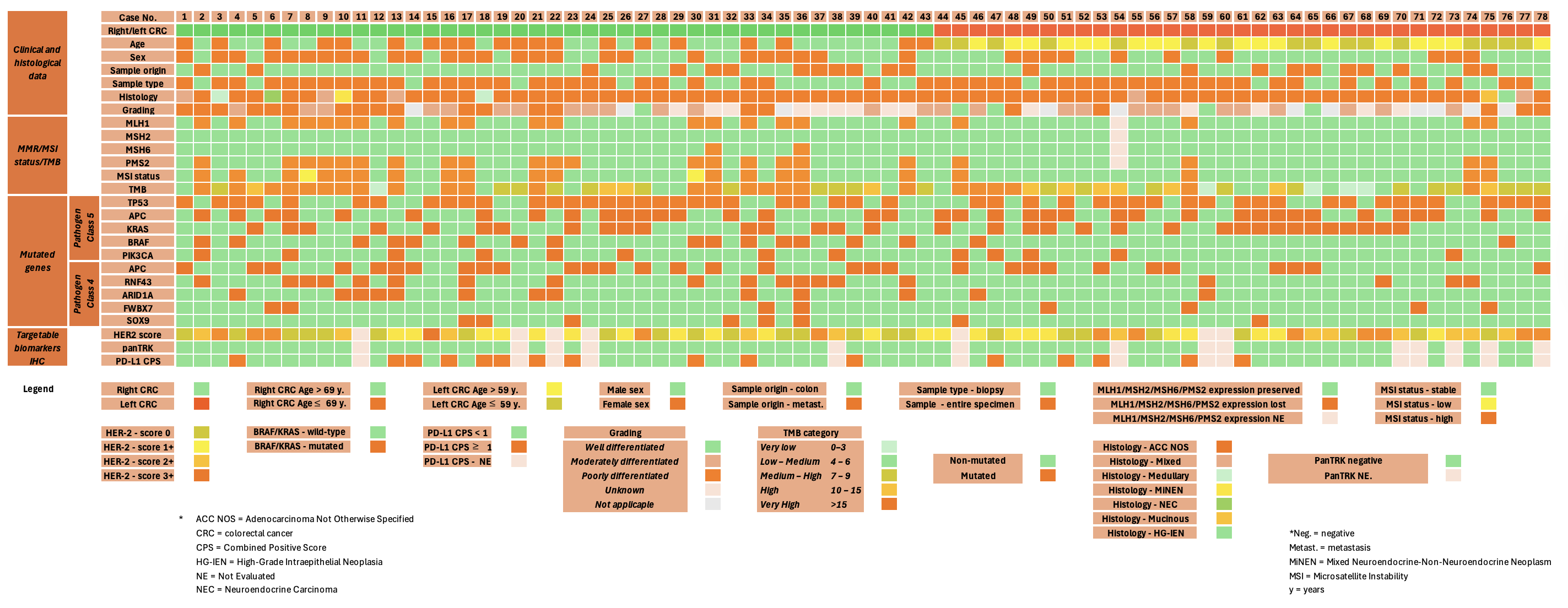

Supplement: Supplementary file 1 [file cancers-18-00666-s001.zip › Figure S2. Heatmap.png]
